# Antarctic Circumpolar Current creates a boundary limiting the meridional dispersal of bacteria in the Southern Ocean

**DOI:** 10.1002/mlf2.70094

**Published:** 2026-08-05

**Authors:** Siqi Lin, Yanru Dang, Yu Wang, Ninghua Liu, Chunyang Li, Pingyi Li, Xiying Zhang, Xiulan Chen, Yu Xin, Li Liao, Andrew McMinn, Yuzhong Zhang, Qi‐Long Qin

**Affiliations:** ^1^ State Key Laboratory of Microbial Technology, Shandong Mental Health Center Shandong University Qingdao China; ^2^ MOE Key Laboratory of Evolution and Marine Biodiversity, Frontiers Science Center for Deep Ocean Multispheres and Earth System & College of Marine Life Sciences Ocean University of China Qingdao China; ^3^ Joint Research Center for Marine Microbial Science and Technology Shandong University and Ocean University of China Qingdao China; ^4^ Key Laboratory of Polar Science, Ministry of Natural Resources Polar Research Institute of China Shanghai China; ^5^ Institute for Marine and Antarctic Studies University of Tasmania Hobart Australia; ^6^ Laboratory for Marine Biology and Biotechnology Qingdao Marine Science and Technology Center Qingdao China; ^7^ Marine Biotechnology Research Center, State Key Laboratory of Microbial Technology Shandong University Qingdao China

## Abstract

As the world's largest ocean current in terms of volume transport, the Antarctic Circumpolar Current (ACC) significantly affects the movement of heat and water in the Southern Ocean. However, the impact of the ACC on the biogeographic distribution of marine bacteria is unknown. Here, by sampling and investigating the bacterial communities from surface seawater samples surrounding Antarctica and across the ACC region, we show that the ACC acts as a barrier limiting the dispersal of bacteria through it. This dispersal limitation, which reduces the communication between bacteria near Antarctica and foreign bacteria, will possibly be impacted by future climate change.

Marine microorganisms play crucial roles in biogeochemical cycling and energy flow in the world's oceans^1^, ^2^, ^3^. However, global climate change will likely affect future species distributions and ecological functions of these microorganisms^4^. Polar regions are particularly susceptible to the impacts of global warming. For instance, the melting rate of both Arctic and Antarctic sea ice has noticeably accelerated in recent years^5^. This will significantly impact the distribution and function of marine microorganisms in polar seawater. The Antarctic region consists of the Antarctic continent surrounded by the Southern Ocean (SO), serving as a crucial link between the Atlantic, Pacific, and Indian oceans^6^, ^7^. Due to its openness and close interaction with adjacent oceans, SO is accompanied by the Antarctic Circumpolar Current (ACC), one of the most violent and intense currents in the world driving the global circulation system.

The ACC extends from approximately 35°S to 65°S. It has traditionally been considered to contain five main fronts from north to south: the Subtropical Front (STF), the Subantarctic Front (SAF), the Polar Front (PF), the southern ACC Front (sACCF), and the southern Boundary front (sBdy), separating the subtropical waters from the polar waters^8^. ACC coincides with the belt of westerly wind that is considered as the main driving force forming the ACC^9^. This westerly wind has a strong influence on the transport of heat as well as the biogeographic distribution of airborne bacteria over the SO^10^, ^11^. Similarly, the ACC is able to restrict the meridional flow of water and energy in the SO, and together with the circumpolar atmospheric vortex, can mitigate the effects of climate change on Antarctic environments^12^. The ACC has long been considered as a major barrier to the dispersal of the majority of marine eukaryotes in the SO, such as fish and invertebrates^13^. However, as the ACC restricts the movement of heat and water in the SO, how it will shape the geographical distribution of microbes in the SO seawater remains unknown.

In this study, we assessed the structure, assembly process, and biogeographic distribution pattern of microbial communities in 45 surface seawater samples collected across the ACC (42°S–70°S). Based on the sampling location and front position, 45 samples were grouped into three regions: the Circum‐Antarctic Region (CAR, 28 selected samples from ZH_02 to ZH_56 distributed on and within the sACCF), the Westerly Wind Region (WWR, 9 samples including ZH_00, ZH_01, and ZH_57 to ZH_63 across all fronts), and the Northern Boundary of Southern Ocean (NBSO, 8 samples from ZH_64 to ZH_71 distributed outside of the STF) (Figure 1A).

**Figure 1:**
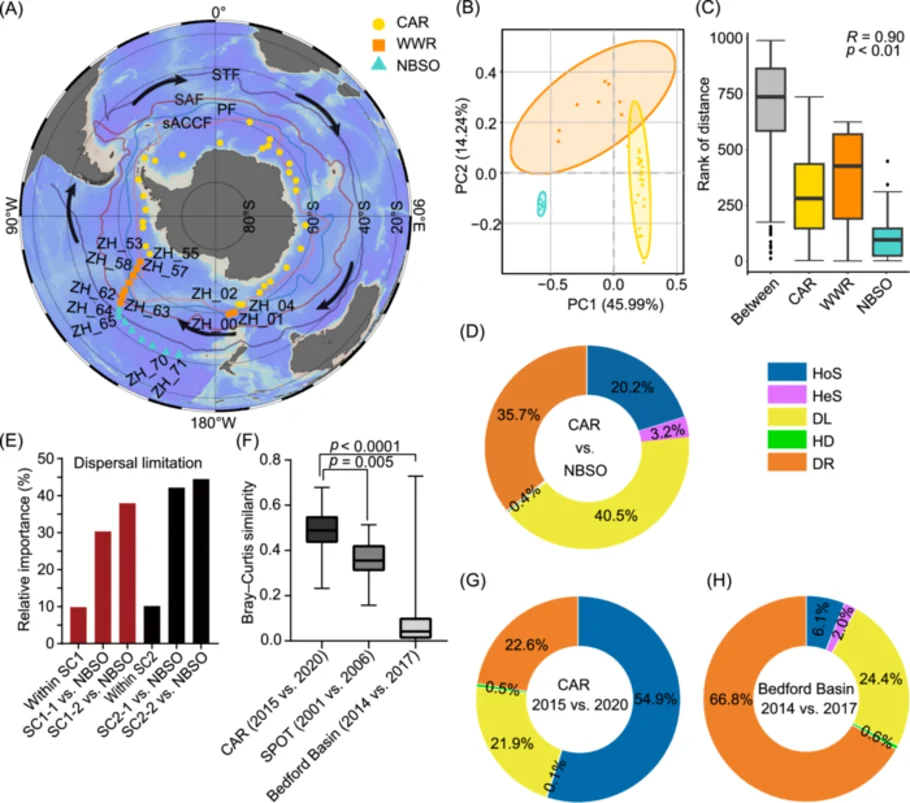
Sample location, community similarity, and ecological processes driving community assembly among three groups in the Southern Ocean. (A) Geographical location of sample stations. The colored circle represents groups with different latitude ranges: CAR (Circum‐Antarctic Region, yellow), WWR (Westerly Wind Region, orange), and NBSO (Northern Boundary of Southern Ocean, cyan). For clarity, only the key sample names are shown. The fronts shown are, from north to south, the Subtropical Front (STF), the Subantarctic Front (SAF), the Polar Front (PF), and the southern ACC Front (sACCF). (B) Principal coordinates analysis (PCoA) of the community structure. (C) Analysis of similarities (ANOSIM) based on the Bray–Curtis distance among the three groups. The *R*‐value and *p*‐value indicate the ANOSIM statistic and its significance level, respectively. (D) Relative importance of ecological processes in driving the community assembly between groups CAR versus NBSO using the iCAMP framework. HoS, homogeneous selection; HeS, heterogeneous selection; DL, dispersal limitation; HD, homogenizing dispersal; and DR, drift. (E) Relative importance of DL within and between the two CAR sub‐categories (SC1 and SC2) versus NBSO. (F) Microbial community similarity between different years in CAR, San Pedro Ocean Time Series (SPOT), and Bedford Basin. Differences between groups were statistically evaluated using a paired *t*‐test. (G, H) Relative importance of ecological processes in driving the community assembly between different years in CAR (G) and Bedford Basin (H). iCAMP, infer community assembly mechanisms by phylogenetic‐bin‐based null model analysis.

At the class level, taxonomic profiling of bacterial communities showed that WWR and NBSO groups had a relatively higher abundance of unidentified *Marinimicrobia* and *Verrucomicrobiae* than that in CAR (Figure S1A). At the genus level, distinct abundances of specific genera in each group were detected using linear discriminant analysis effect size (LEfSe) analysis (LDA > 3.0, *p* < 0.05) (Figure S2). The genera *Polaribacter* and *Porticoccus* were especially abundant in CAR compared to WWR and NBSO. The relative abundance of genus *Vicingus* in WWR and genus *Magnetovibrio* in NBSO was higher than that in CAR (Figure S1B). Generally, these results highlighted that each group had specific abundant genera, indicating distinct bacterial community compositions across different groups of the SO.

The index of effective operational taxonomic unit (*N*_eff_ = e^Shannon index^) was used to evaluate the alpha diversity of each group^14^. The N_eff_ values were significantly different among the three groups (ANOVA, *p* < 0.001, *F* = 83.4), with the NBSO having the highest value (average *N*_eff_ of 113.7) and the CAR having the lowest value (average *N*_eff_ of 46.1) (Figure S3). This suggested that three groups had different community‐wide biodiversity, with samples from CAR having the lowest one. Beta diversity analyses were performed to assess the heterogeneity of communities among the three groups. The principal coordinates analysis (PCoA) demonstrated that samples were clustered into three groups based on the sample locations (Figure 1B). In addition, the analysis of community similarity (ANOSIM) further confirmed a significant difference (*R* = 0.9, *p* < 0.01) in bacterial communities among different groups (Figure 1C), supporting the clustering of bacterial communities based on the three distinct groups. Taken together, both alpha and beta diversity analyses demonstrated significant differences in microbial communities between the three front‐based groups.

The infer community assembly mechanisms by phylogenetic‐bin‐based null model analysis (iCAMP) framework was used to investigate the ecological drivers of community assembly within and between three groups^15^. Five processes, homogeneous selection (HoS), heterogeneous selection (HeS), dispersal limitation (DL), homogenizing dispersal (HD), and drift (DR), were calculated and compared to evaluate their relative contributions to the community turnover. The results showed that HoS (belonging to a deterministic process) was the dominant process in bacterial community assembly within each group (Figure S4). However, when considering the community turnover between groups, the relative importance of stochastic processes, including DL, HD, and DR, increased greatly (51.9%–56.6% for within groups and 64.1%–76.6% for between groups). Especially, for the community turnover between CAR and NBSO, the DL was the most important process and its contribution (40.5%) was much higher than that of HoS (20.2%) (Figure 1D). This implies that the ACC can present a barrier limiting the dispersal of strains between CAR and NBSO regions.

As DL is related to the geographical distance between samples, how distance would affect DL contribution within CAR as well as between CAR and NBSO is further investigated. For this purpose, two sub‐category (SC) samples with oppositely oriented positions and similar distance in CAR were selected (Figure S5). The contributions of DL to community assembly within two sub‐categories in CAR as well as between each sub‐category and NBSO were then compared (Figure 1E). DL's contribution to community assembly between SC1‐2 and NBSO increased by approximately 4 times compared with that within sub‐category 1 (SC1‐1 versus SC1‐2); even their average distances were similar (5679 versus 5988 km). The contribution of DL within sub‐category 2 (SC2‐1 versus SC2‐2, 10.1%) was nearly a quarter of SC2‐1 versus NBSO (42.2%) and SC2‐2 versus NBSO (44.5%) (Figure 1E). Although the contribution of DL to the community assembly of SC2‐1 versus NBSO and SC2‐2 versus NBSO was similar, the average distance of SC2‐1 versus NBSO (3639 km) was ~53% shorter than that of SC2‐2 versus NBSO (7871 km). Notably, all large values of DL were observed in geographical ranges crossing the ACC, suggesting that the ACC could form a barrier limiting the entrance of foreign colonizers into Antarctic ocean and play a major role in governing bacteria distribution and community turnover in the SO surface water.

The effect of the ACC on microbial potential functions among different groups was further investigated using the metagenomic data of eight samples from three groups. The variations in community functions among three groups were compared based on the relative abundance of functional genes assigned to KEGG Orthology (KO) as well as involved in nitrogen, phosphorous, and sulfur metabolisms. The principal components analysis (PCA) showed that except for nitrogen metabolism, samples were grouped into three clusters based on sample locations for all the functional categories (Figure S6). Each group had specific high‐abundance genes. For example, genes encoding iron (III) transport system ATP‐binding protein (K02010) were enriched in CAR (Figure S7), consistent with the iron limitation in the SO^7^, ^13^. The distinct functional profiles of different groups suggest that ACC can also affect the biogeography of microbial functions in the SO surface water.

The taxonomic and functional profiling and alpha/beta diversity analyses showed that the three groups based on latitude had different bacterial community compositions as well as specific dominant taxa and functions. The stochastic ecological process, mainly DL, primarily drove the community turnover between groups, especially between CAR and NBSO. DL and consequent geographic isolation are considered normal and vital mechanisms in promoting community variations^16^, ^17^, ^18^. Biological connectivity and communication can be reduced by DL that produces barriers between different locations. The clear differences in bacterial community and functions between the three groups, especially between CAR and NBSO, suggest that the ACC provides a boundary limiting the meridional dispersal of bacteria in the SO.

In the SO, different fronts delimit the boundaries between different water masses with distinct environmental characteristics. Each front is considered to represent a sharp barrier to the meridional dispersal and movement of water masses^6^, ^19^. The observed bacterial dispersal barriers of the ACC are likely to be related to different fronts that limit the meridional transport of seawater in the SO. Consistent with the meridional barrier of water mass movements, the concentration of dissolved inorganic nutrients (nitrate and nitrite, phosphate, and silicate) changed sharply from the CAR to NBSO region (Figure S8A–C). For example, the silicate concentration decreased from 40.9 µmol/l in CAR to nearly undetectable levels in NBSO, while the microbial cell number and dissolved oxygen of the three groups did not vary significantly (Figure S8D,E). The abrupt, not gradual, change of nutrient concentrations further supported the barrier ability of ACC. Due to the availability of sampling time, all the samples in this study were collected during the polar day of the Antarctic region without obvious seasonal changes. Seasonal changes could affect the bacterial communities, environmental factors as well as oceanic hydrodynamics in the SO. Therefore, how seasonal changes would influence the microbial biogeographic pattern is to be investigated.

Community assembly within CAR was dominated by HoS (relative importance of 45.8%) (Figure S4). Meanwhile, environmental factors are relatively consistent in CAR. These characteristics suggest that ACC may serve as a mixing force in the SO, especially at the southern boundary of ACC, making environmental factors and microbial communities homogeneous. Besides functioning as a barrier to limit the meridional transport of marine bacteria, ACC also facilitates communication between microbial communities within the same front. Thus, it is likely that the interannual similarity of seawater microbial communities in CAR would be greater than in areas without an obvious dispersal barrier. The similarity between microbial communities from different years in CAR was calculated and compared with that from seawater in the Bedford Basin of Atlantic Ocean and the San Pedro Ocean Time Series (SPOT) site of Pacific Ocean. The results showed that the CAR microbial communities, taken at a 5‐year interval, were more similar to each other than to those from the Bedford Basin seawater (3‐year interval) and SPOT (5‐year interval) (Figure 1F). Ecological driver analyses showed that HoS dominated the community assembly in CAR between different years, while DR dominated the community assembly between different years in Bedford Basin (Figure 1G,H). This suggests that the ACC can conserve the integrity of bacterial communities in seawater surrounding the Antarctic over time.

The results presented here show that seawater from distinct latitudinal regions in the SO has different biodiversity and that DL is the main force driving the community turnover between different areas. This DL is considered to be conferred by the different fronts in ACC. We propose that the ACC creates a boundary limiting the meridional dispersal of marine bacteria that results in the observed biogeographic pattern of bacterial community and function. This barrier could limit the migration of foreign microorganisms and maintain the relatively pristine nature of marine bacteria near Antarctica. Evidences suggest that the SO is observed to become warm, and the ACC tends to move southward^9^, ^20^. This study has laid a foundation for evaluating the potential influence of future ACC change on microbial communities in the SO.

## ETHICS STATEMENT

This study did not involve human subjects and animals.

## Supporting information

Supporting File 1.

## Data Availability

Sequencing data of 16S rRNA gene and metagenome are available in the National Genomics Data Center (NGDC, https://ngdc.cncb.ac.cn/) database of China with GSA ID CRA016334 and CRA033109.
