# Supplementary material for: Antarctic Circumpolar Current creates a boundary limiting the meridional dispersal of bacteria in the Southern Ocean
Source: mLife. 2026 Aug 5;5(4):520–4. doi: 10.1002/mlf2.70094 (PMC13439544; doi:10.1002/mlf2.70094)
Supplement: Supplementary file 1 [file MLF2-5-520-s001.docx]

**Supporting Information for**

**Antarctic Circumpolar Current creates a boundary limiting the meridional dispersal of bacteria in the Southern Ocean**

**This file includes:**

Materials and Methods, and Supplementary Figures Fig. S1 to S8.

**Materials and Methods**

**Sample collection**

A total of 45 surface seawater samples (~ 4 m depth) were collected across the Southern Ocean, spanning latitudes from 42 °S to 70 °S during the 37th Chinese National Antarctic Research Expedition on R/V Xue Long2, from December 7, 2020, to April 9, 2021. The positions of all sampling points were created using Ocean Data View software (Fig. 1A). Seawater samples were initially filtered through a bilayer 50 µm pore-size silk yarn to remove large particles and then filtered onto 0.22 µm pore-size polycarbonate membranes to collect the microbial cells. The filtered membranes were carefully stored in sterile tubes and frozen at -80 °C in the lab until nucleic acid extraction. The *in situ* seawater temperature, pH and DO were detected using the OceanPack system (SubCtech, Germany). The dissolved organic carbon (DOC) content was measured by a TOC analyzer (TOC-L, Shimadzu, Japan) [1], while nutrient contents, including nitrate (NO_3_^−^), nitrite (NO_2_^−^), phosphate (PO_4_^3-^), and silicate (SiO_3_^2-^ ) were measured by spectrophotometric and colorimetric analyses [2].

**DNA extraction, amplicon sequencing and analyses**

Total DNA were extracted from the membranes with PowerWater® DNA Isolation Kits (MOBIO, USA) according to the manufacturer’s protocols. The acquired DNA of each sample were used as templates to amplify the V3-V4 region of the bacterial 16S ribosomal RNA (rRNA) gene by PCR [3]. Amplicon sequencing was conducted using Illumina MiSeq technology subsequently for each sample at Majorbio Bio-Pharm Technology Co. Ltd. (Shanghai, China).

Trimmed and merged reads received from Illumina sequencing were imported, quality controlled, de-replicated, and allocated to operational taxonomic units (OTUs) using a 0.97 identity by Qiime2 [4]. An OTU table was formed by mapping each reads to a representative OTU sequence. To study the taxonomic relationship, each representative OTU sequence was assigned taxonomy with the SILVA database [5, 6]. After removing OTUs belonging to mitochondria, chloroplast, and Archaea, the OTU table was processed to ensure equal numbers of reads per sample (64,650) using the vegan package in R and subsequently used to analyze the bacterial community compositions and relative abundance of each sample.

The analyses of bacterial alpha diversity of each sample and beta diversity between groups, including principal coordinates analysis (PCoA) and one-way analysis of community similarity (ANOSIM) were conducted by the R package vegan. Rooted and unrooted phylogenetic tree were constructed by Fasttree based on 16S rRNA gene information after the representative OTU sequences were aligned with muscle [7, 8]. Based on the relative abundance of each OTU, a quantitative framework iCAMP was employed to investigate the driving force forming the diversity within and between groups using the R package icamp [9]. The geographical distances between the two sampling locations were calculated by Python script with the geopy module. Linear discriminant analysis effect size (LefSe) was performed to explore the indicator OTU in each group [10].

The OTU table of CAR microbial communities sampled in 2015, the OTU table of the phytoplankton community in coastal seawater of Bedford Basin located at Northwest Atlantic, the OTU table of bacterioplankton community in seawater of the San Pedro Ocean Time Series (SPOT) site located at Northwest Pacific, and the environmental factors of two sites [11-13] were also collected for analyses of community Bray-Curtis similarities and assembly processes between different years.

**Metagenomic sequencing and Analyses**

Metagenomic shotgun sequencing libraries were constructed and sequenced at Shanghai Biozeron Biological Technology Co. Ltd. The libraries were sequenced on an Illumina MiSeq platform and 150 bp paired-end reads were generated. Approximately 50 Gbp raw sequencing data were obtained for each sample. Raw sequence reads underwent quality trimming using Trimmomatic (v0.38) to remove adaptor contaminants and low-quality read. Megahit was used to concatenate clean reads sequences. Prodigal v2.6.3 was performed for predicting open reading frames (ORFs) in concatenated sequences. Then a non-redundant gene set of all ORFs with identity set to 95% and coverage set to 90% was constructed by CD-HIT v4.8.1.

The abundance of DNA based genes in each sample was estimated by the number of mapped reads and normalized TPM (mapped reads per million)=10^6^ × (mapped reads/gene length)/sum of (mapped reads/gene length). BWA was used to map reads to a non-redundant gene set and calculate the TPM value for each gene in each sample. All protein sequences were aligned in nitrogen, phosphorus, and sulfur database by blastp and performed KO annotation by eggNOG-mapper. The abundance of genes annotated to KO numbers and involved in nitrogen, phosphorus and sulfur cycling in each sample were further analyzed by PCA using 0.9 confidence interval [14-16]. The Random Forest method is used to select KO numbers with differential gene expression between groups and draw heatmaps by R.

Fig. S1. Taxonomic composition of each sample at the class (A) and genus (B) level.

Fig. S2. Linear discriminant analysis effect size (LEfSe) of three groups at the genus level. The top OTUs in the LDA score ranking of the three groups were illustrated.

Fig. S3. The Neff (e^Shannon^ index) value of three groups.

**Fig. S4.** The relative importance of different ecological processes in driving community assembly within each group.

Fig. S5. Map showing two sub-categories of the opposite sub-categories in CAR. The red dots belong to SC1 while the black dots belong to SC2.

Fig. S6. Principal components analyses (PCA) of the functional genes assigned to KOs (A), as well as involved in nitrogen (B), phosphorous (C) and sulfur (D) metabolisms in three groups.

**Fig. S7.** The relative abundance of differential KOs among three groups. The color scale of the heatmap represents the gene abundance value using the z-score.

Fig. S8. (A-D) Concentrations of nitrate and nitrite, phosphorus, silicate and DO in each sample of three groups respectively. (E) Flow cytometric counts of microbial cells.

**References**

1. Spyres G, Nimmo M, Worsfold PJ, Achterberg EP, Miller AEJ. Determination of dissolved organic carbon in seawater using high temperature catalytic oxidation techniques. Trac-Trend Anal Chem. 2000;19(8):498-506.

2. Hansen HP, Koroleff F. Determination of nutrients. Methods of Seawater Analysis. Wiley Online Library; 1999.

3. Tiveljung A, Backstrom J, Forsum U, Monstein HJ. Broad-range PCR amplification and DNA sequence analysis reveals variable motifs in 16S rRNA genes of Mobiluncus species. APMIS. 1995;103(10):755-63.

4. Hall M, Beiko RG. 16S rRNA Gene Analysis with QIIME2. Methods Mol Biol. 2018;1849:113-29.

5. Quast C, Pruesse E, Yilmaz P, Gerken J, Schweer T, Yarza P et al. The SILVA ribosomal RNA gene database project: improved data processing and web-based tools. Nucleic Acids Res. 2013;41(Database issue):D590-6.

6. Yilmaz P, Parfrey LW, Yarza P, Gerken J, Pruesse E, Quast C et al. The SILVA and "All-species Living Tree Project (LTP)" taxonomic frameworks. Nucleic Acids Res. 2014;42(Database issue):D643-8.

7. Edgar RC. MUSCLE: multiple sequence alignment with high accuracy and high throughput. Nucleic Acids Res. 2004;32(5):1792-7.

8. Price MN, Dehal PS, Arkin AP. FastTree 2--approximately maximum-likelihood trees for large alignments. PLoS One. 2010;5(3):e9490.

9. Ning D, Yuan M, Wu L, Zhang Y, Guo X, Zhou X et al. A quantitative framework reveals ecological drivers of grassland microbial community assembly in response to warming. Nat Commun. 2020;11(1):4717.

10. Segata N, Izard J, Waldron L, Gevers D, Miropolsky L, Garrett WS et al. Metagenomic biomarker discovery and explanation. Genome Biol. 2011;12(6):R60.

11. Cram JA, Chow CE, Sachdeva R, Needham DM, Parada AE, Steele JA et al. Seasonal and interannual variability of the marine bacterioplankton community throughout the water column over ten years. ISME J. 2015;9(3):563-80.

12. Robicheau BM, Tolman J, Bertrand EM, LaRoche J. Highly-resolved interannual phytoplankton community dynamics of the coastal Northwest Atlantic. ISME Commun. 2022;2(1):38.

13. Wang ZB, Sun YY, Li Y, Chen XL, Wang P, Ding HT et al. Significant bacterial distance-decay relationship in continuous, well-connected southern ocean surface water. Microb Ecol. 2020;80(1):73-80.

14. Tu Q, Lin L, Cheng L, Deng Y, He Z. NCycDB: a curated integrative database for fast and accurate metagenomic profiling of nitrogen cycling genes. Bioinformatics. 2019;35(6):1040-8.

15. Zeng J, Tu Q, Yu X, Qian L, Wang C, Shu L et al. PCycDB: a comprehensive and accurate database for fast analysis of Phosphorus cycling genes. Microbiome. 2022;10(1):101.

16. Yu X, Zhou J, Song W, Xu M, He Q, Peng Y et al. SCycDB: a curated functional gene database for metagenomic profiling of Sulphur cycling pathways. Mol Ecol Resour. 2020;21(3):924-40.
